# Understanding muscle-immune interactions in adolescent idiopathic scoliosis: a feasibility study

**DOI:** 10.1186/s40814-017-0193-0

**Published:** 2017-12-05

**Authors:** Srikesh Rudrapatna, Devin Peterson, Paul Missiuna, Ishan Aditya, Brian Drew, Nicola Sahar, Lehana Thabane, M. Constantine Samaan

**Affiliations:** 1 Department of Pediatrics, McMaster University, 1280 Main Street West, HSC-3A57, Hamilton, Ontario Canada; 2 Department of Pediatric Surgery, Division of Orthopedics, McMaster University, Hamilton, ON Canada; 3 Department of Health Research Methods, Evidence and Impact, McMaster University, Hamilton, Ontario Canada; 4 Division of Pediatric Endocrinology, McMaster Children’s Hospital, Hamilton, ON Canada; 5 Department of Anesthesia, McMaster University, Hamilton, ON Canada; 6 Centre for Evaluation of Medicines, Hamilton, ON Canada; 7 Biostatistics Unit, St Joseph’s Healthcare-Hamilton, Hamilton, ON Canada

**Keywords:** Adolescent idiopathic scoliosis, Immunometabolism, Macrophage, Paraspinal muscle, Feasibility study, ICONS study

## Abstract

**Background:**

Adolescent idiopathic scoliosis (AIS) is the most common form of scoliosis in children, and its cause remains unknown. The Immune-metabolic CONnections to Scoliosis (ICONS) Study was designed to elucidate the potential mechanisms by which immune system-paraspinal muscle crosstalk contributes to the development of AIS. In this report, we document the evaluation of ICONS Study feasibility.

**Methods:**

This study was conducted at a tertiary pediatric academic center in Hamilton, Ontario, Canada. We included boys and girls, aged 10–17 years with a diagnosis of AIS requiring corrective spinal surgery. Exclusion criteria included patients on high-dose steroids, immunosuppressive therapy, anti-thrombotic medications, those with an active infection for 15 days before participation**,** autoimmune disease, pregnancy, and patients who were unwilling to consent.

Pre-determined feasibility criteria included permission to approach participants and recruitment rates of 80%, consenting of at least 80% of participants to provide biological samples, 90% or higher case report form and questionnaire completion, resources to be sufficient in at least 80% of recruitments, and the ability to successfully collect and process 80% or more of the biological samples needed for this study.

**Results:**

Between August 2013 and October 2014, we identified 32 potential participants with AIS, but had the resources to approach only 16, of which 12 (75%) agreed to be approached by the research team, and all consented to participate. Of the 12 participants recruited, 11 questionnaire packages and muscle biopsies (91.7% for each objective) were collected, while other biological samples (serum, plasma, whole blood for DNA and RNA processing, urine) were collected from all participants.

**Conclusions:**

The ICONS study protocols and procedures are feasible. However, recruitment rates were less than predicted. For the full study, we plan on prolonging the recruitment phase and the inclusion of additional centers to achieve recruitment targets.

## Strengths and limitations of this study

- Study procedures have been assessed for feasibility.
- The study demonstrated the feasibility of building collaborations with clinical services essential for study conduct.
- This feasibility study has informed certain modifications to the procedures implemented in the Immune-metabolic CONnections to Scoliosis (ICONS) Study. This ensures successful participant recruitment, data acquisition, and sample collection and processing.
- This feasibility study informs translational researchers of the elements that need to be considered when designing translational research studies.
- This study is potentially limited by the use of tools that rely on self-reported data, and are subject to inherent biases. However, utilizing validated tools minimized the impact of these biases.
- Another limitation is the cross-sectional nature of the study. Due to the nature of the study procedures including tissue biopsies, it is not possible to use another study design at this stage.

## Background

Adolescent idiopathic scoliosis (AIS) is the most common form of spinal curving in youth, and occurs mainly in girls. While the cause of AIS remains unknown, it is likely that it is caused by a complex interaction of genes, the nervous system, hormones, metabolic dysfunction, skeletal abnormalities, biochemical factors, as well as environmental and lifestyle factors [1–9]. In some cases, AIS is associated with several comorbidities including pain, pulmonary hypertension, and cosmetic effects, which increase the burden of this condition on the affected youth although the impact of AIS on these comorbidities and the justification for surgery based on them has been debated [10].

Several paraspinal tissues may play a role in AIS, but the exact contribution of different tissues to the genesis of AIS are not fully understood. While paraspinal muscles are important in maintaining spinal stability and mobility, it is unclear if they contribute to the initiation or propagation of spinal curvature, or if their phenotype is secondary to scoliosis. Regardless, studying muscle profile in AIS may lead to better understanding of the mechanisms involved in the development of scoliosis.

One of the important phenotypic features noted in paraspinal muscle is the infiltration of macrophages and fibrosis, which begs the question of the role of immune system-muscle interactions in AIS etiopathogenesis [11–14]. While the presence of macrophages in muscle has been commented on briefly in studies that have described these findings, no detailed phenotyping of these macrophages and their contribution to muscle pathophysiology has been undertaken.

The Immune-metabolic CONnections to Scoliosis (ICONS) Study was designed to investigate the mechanisms of immune-muscle crosstalk in AIS. The protocol of the study was recently published [15].

The complex nature of study procedures involve consenting patients to obtain biological samples for the study, the attendance of spinal surgeries to collect tissue and other biological samples, and immediate processing of tissue samples in the operating room. It was unclear whether patients would be willing to consider participating in the study, considering the invasiveness of some of the study procedures. In addition, the feasibility of collecting high-quality biological samples needed to be determined in the clinical setting.

The final goal of the study is to recruit 120 subjects, therefore determining if timelines are realistic would be important. When completed, the ICONS study will be the largest study in the field of AIS immunometabolism undertaken so far, so feasibility measures are critical prior to committing resources to the full study. We did not identify any previous scoliosis studies that reported on the details of study procedures in a similar setting.

Our goal in this paper is to report on the feasibility of the ICONS study clinical processes and laboratory procedures, and indicate subsequent modifications based on these results. As studies of this nature are relatively rare in the fields of pediatric orthopedic and translational research, our goal was to establish the feasibility of the study procedures before launching the full study.

## Methods

### Setting and participants

The ICONS Study is being conducted at McMaster Children’s Hospital, a tertiary pediatric academic center in Hamilton, Ontario, Canada. In the recently published protocol, a sample size of 120 participants recruited over 5 years was deemed sufficient to detect significant differences in the analyses [15].

### Recruitment

**Fig. 1:**
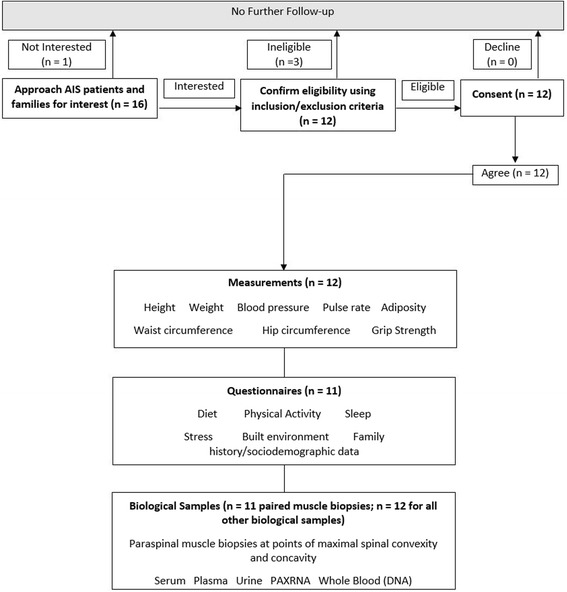
ICONS Study recruitment and study procedures

Patients are recruited from the Orthopedics Clinic at the Hospital. The Hamilton Integrated Research Ethics Board approved the study. Figure 1 reports study recruitment procedures.

### Inclusion criteria

We included boys and girls, 10–17 years old, who are diagnosed with AIS and need corrective spinal surgery.

### Exclusion criteria

For this specific study, patients with non-idiopathic scoliosis, such as congenital scoliosis and scoliosis due to neuromuscular disorders, skeletal dysplasia, and syndromes were excluded. Other reasons for exclusion included patients on high dose steroids (i.e. above the physiological substitution doses of 6–8 mg/m^2^/day), immunosuppressants, or anti-thrombotic medications 15 days prior to surgery. In addition, exclusion criteria included smoking, active infections, autoimmune disease, pregnancy, and patients who were unable or unwilling to consent, or when anthropometric measurements were not possible [15].

### Consent

The healthcare team involved in the care of potential participants obtained verbal consent from participants to be approached by the study team. If the participant and parents approve, the researcher would then introduce the study and answer their questions.

If there is agreement to participate, the youth or their parent/guardian will sign the consent forms. Participants 16 years or older signed their own consent forms, while those 7–15 years of age signed an assent form while their parent/guardian signed the consent forms. Unique identifiers were assigned to allow data anonymization shortly after data and biological sample collection to ensure confidentiality.

### Collection of participant data

Standardized tools were utilized to collect data regarding nutrition [16], physical activity [17], sleep [18], mental health [19, 20], and neighborhood walkability [21]. In addition, sociodemographic data including age, sex, ethnicity, parental and participants’ education, parental profession, family income, social and family history, past medical and surgical history, current medications, pregnancy and birth history, and menstrual history in girls were collected. Anthropometric and clinical measures collected included height (cm), weight (kg), from which Body Mass Index (BMI, kg/m^2^) and BMI percentile calculations were derived. Waist circumference (cm), hip circumference (cm), blood pressure (mmHg), heart rate (beats per minute), and grip strength (kg) using a dynamometer were measured. The fat mass percentage was tested using bioelectrical impedance (Tanita Corporation, Arlington Heights, USA) [22]. Data were managed using Research Electronic Data Capture (REDCap) tools hosted at McMaster University [23].

### Biological sample collection and processing

**Fig. 2:**
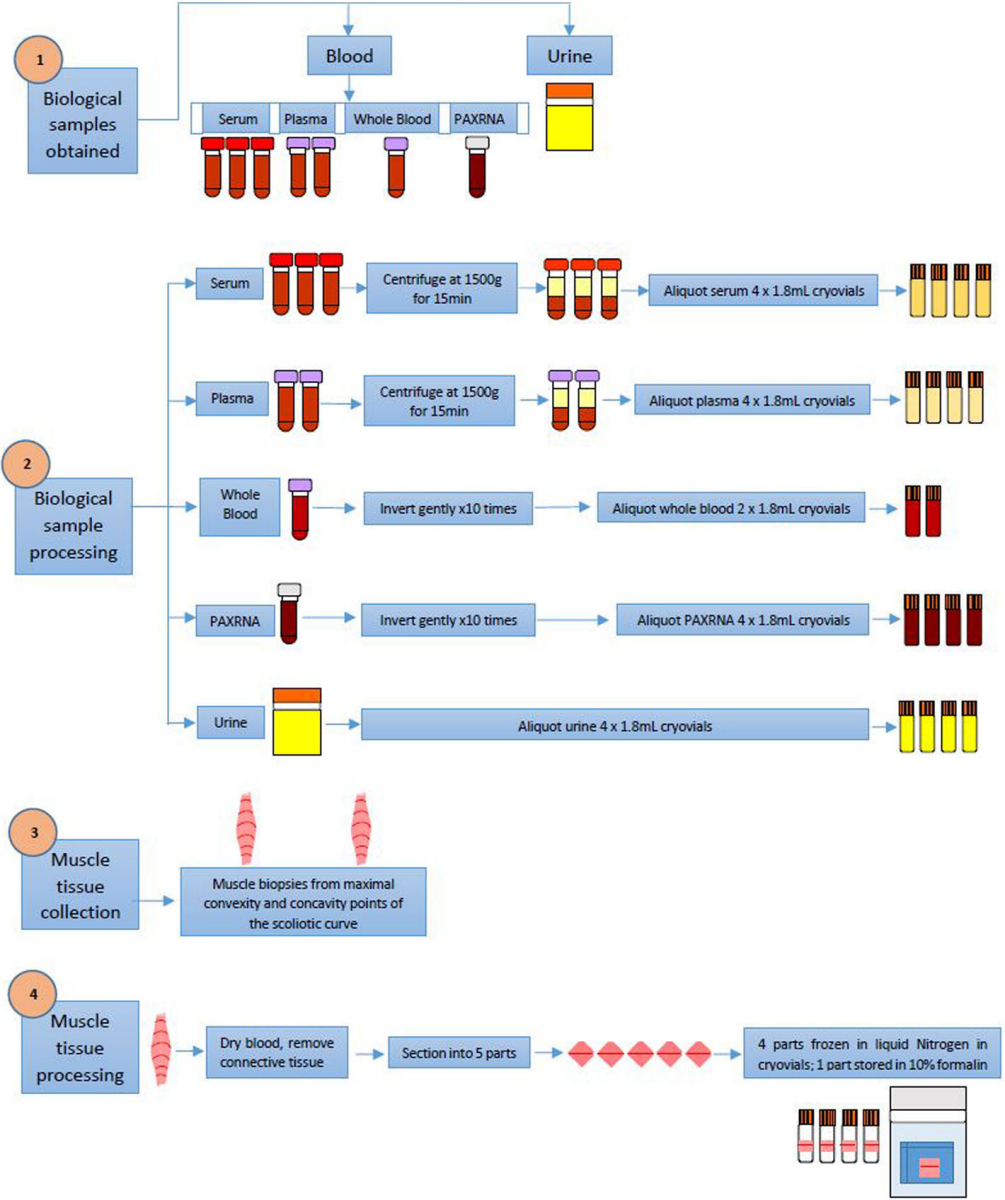
Biological sample processing flow chart

The biological sample processing protocol is presented in Fig. 2, and reported in detail in the published study protocol [15]. Liquid biopsies including blood and urine samples were collected after a midnight fast from central lines and urinary catheters in the operating room, respectively. All samples were processed and stored within two hours of collection. Part of the muscle biopsies was snap-frozen in liquid nitrogen, and another part was preserved in formalin immediately after collection. The formalin-treated samples were then embedded in paraffin and stored until further processing.

### Blood samples

*Serum:* Fasting serum samples were isolated from blood samples collected into anticoagulant-free tubes (BD Life Sciences, Ontario, Canada). The samples were centrifuged for 15 min at 1500*g*, at room temperature. Samples were aliquoted and stored at −80 °C until further processing.

*Plasma:* Fasting plasma samples were obtained from EDTA-treated blood samples by centrifuging for 15 min at room temperature at 1500*g.* Samples were loaded into cryovials for storage at −80 °C until further processing.

*RNA samples:* PAXRNA tubes (BD Life Sciences, Ontario, Canada) were used to collect and preserve leucocyte RNA from whole blood. Samples were processed according to manufacturer’s instructions and then aliquoted into cryovials and stored at −80 °C until further use.

*DNA samples:* In the original protocol, we planned on using buffy coats to obtain DNA [15]. Based on challenges in standardizing this approach, an alternative approach of obtaining whole blood samples from EDTA-containing vacutainer tubes (BD Life Sciences, Ontario, Canada) was adopted. These samples were aliquoted into cryovials and samples were stored at −80 °C until further processing.

### Urine samples

Fasting urine samples were collected into 90-mL containers. The samples were then aliquoted into cryovials and frozen at −80 °C until further processing.

### Paraspinal muscle tissue biopsies and processing

Paraspinal muscle biopsies were obtained at points of maximal concavity and convexity of spinal curves. Samples were promptly separated from connective tissue and dried from blood, and then divided into few pieces. These samples were then placed in cryovials and snap-frozen in liquid nitrogen immediately. Soon after, samples were transferred in liquid nitrogen and stored at −80 °C until further processing. The formalin-preserved samples were embedded in paraffin by the Central Pathology Laboratory at Hamilton Health Sciences. These samples will be used in the future in staining and microscopic analyses.

Two important strategies were deployed with muscle tissue collection to preserve immune cell phenotype and integrity. First, these samples were obtained from areas where no cauterization took place, and the samples were collected before injecting adrenaline into muscle. The latter procedure is used to induce vasoconstriction but adrenaline is also known to alter macrophage phenotype [24, 25]. This approach ensures the fidelity of muscle macrophage phenotype in future analyses.

### Measures to determine the feasibility of study procedures

The following criteria were used to indicate the feasibility of study procedures:

1. Participant and parent agreement to be approached and informed about the study in 80% of cases.
2. Of those who agree to be approached, a minimum of 80% will be consented to participate in the study.
3. Of those who agree to participate, case report forms and questionnaires will be completed in a minimum of 90% of participants.
4. Resource utilization including space, researchers’ time, consumables, study protocol testing, and laboratory apparatus use being feasible in 80% of cases.
5. Acquisition and processing of high-quality biological samples in 80% or more of cases.

### Statistical analysis

We evaluated the feasibility of the study procedures in 10% of the total projected study sample size target. Since our target sample size is 120 participants [15], we assessed the feasibility of the study in the first 12 subjects recruited into the study.

Data are reported including the mean (SD) for continuous variables and as a number (percent) for categorical variables. Means and standard deviations were computed using SPSS version 20.0 (IBM Corp., Armonk, New York, USA). We are also reporting medians and ranges of values to provide a more comprehensive view of the participants’ data, as the sample size is small.

## Results

### Participant recruitment

The process by which patients were approached, consented, and asked to provide data is outlined in Fig. 1.

### Participants’ demographics

Over the period from August 2013 to October 2014, while we identified 32 potential eligible participants, we had some logistical challenges in attending the clinics and ended up attending the clinic visit for only 16 of them. This was due to the fact that the students who were involved in the recruitment had scheduling conflicts and were able to only attend a limited number of visits. On one occasion, the patient was rescheduled for their clinic visit by the clinical team, and the research team was not aware of the scheduling change.

Baseline characteristics of the group are outlined in Table 1. Of the 16 subjects, 12 (75%) agreed to be approached by the research team, and all of those approached agreed to participate in the study. Four patients were excluded due to a diagnosis of kyphosis as opposed to AIS (*n* = 1), patient had already undergone a corrective surgery and was having a revision surgery (*n* = 1), patient had multiple sclerosis (*n* = 1), and patient refused to meet the team due to a scheduling conflict (*n* = 1).

**Table 1:** Summary of participant characteristics

Variables	Mean (SD)	Median (range)
Age (years)	14.4 (1.9)	14.4 (11.5–17.5)
BMI (kg/m2)	20.8 (3.0)	20.1 (16.6–26.1)
BMI percentile	58.0 (27.0)	59.3 (18.5–92.5)
Body fat percentage	24.1 (6.2)	23.7 (10.3–35.4)
Cobb angle (degrees)	57.1 (14.8)	56.6 (30.0–78.7)
Pulse rate (bpm)	90.5 (14.9)	88.5 (60.0–122.0)
Systolic blood pressure (mmHg)	121.4 (9.9)	119.5 (108.0–143.0)
Diastolic blood pressure (mmHg)	75.8 (8.3)	76.5 (63.0–88.0)

*Abbreviations*: *SD* standard deviation, *BMI* body mass index, *bpm* beats per minute, *mmHg* millimeters of mercury. All 12 subjects who were approached consented to participating in the study and to providing biological samples (100%)

Most of those recruited were female (*n* = 11; 91.7%). Most participants were Caucasian Europeans (*n* = 9; 75%). The average age of participants was 14.4 ± 1.9 years, and the majority were diagnosed with significant AIS (≥ 40° curves) that required corrective surgery. In most cases, the spinal curve was right-sided (*n* = 10; 83%).

### Feasibility of study procedures

#### Pre-operative recruitment visits

Recruitment procedures were completed within 20 min, including presenting the research study, gathering participant signatures on consent forms and completing anthropometric measures during visit. This indicated that the study procedures are feasible in the clinical setting where these patients are being seen prior to their surgery.

Case report forms reporting height, weight, body fat percentage, blood pressure, and resting heart rate were completed for all subjects (100%). The participants were provided with packages containing the study questionnaires and asked to take these packages home and to return them to the team on the date of surgery. Eleven subjects returned completed questionnaire packages resulting in an overall rate of 91.7% for data collection. One family did not return the package, and further contact attempts in the post-operative period while the patient was in hospital and by phone following discharge were not successful in retrieving the package.

There have been no adverse events during the conduct of the study procedures and biological sample collection.

### Operating room procedures

On the morning of the surgery, the study team attended preoperative multidisciplinary team meetings that included the operating room nursing staff, anesthesia nursing and medical staff, and orthopedic surgeons. The study was highlighted to the attendees who will be present in the operating room during the procedure, and specific requirements for blood, urine, and tissue samples reiterated.

Regarding space utilization in the operating room, a specific location was designated for the research team while patients were being prepared for surgery. The operating room staff set up sterile trays with the needed equipment for biopsy processing.

Once blood, urine, and tissue samples were received from the clinical staff, the research staff completed the sample processing immediately as described above.

Out of all patients who consented to participate, all 12 participants provided serum, plasma, whole blood for DNA and RNA isolation, and urine. For muscle biopsy samples, 11 sets of muscle biopsies were collected by the study team (91.7%). We could not collect muscle tissue samples from one participant due to scheduling difficulties resulting in no trained researchers available to collect and process muscle samples.

### Sample processing in the laboratory

Sample processing flow is demonstrated in Fig. 2. This involved centrifugation of blood samples for serum and plasma isolation as well as aliquoting PAXRNA, whole blood and urine to individual cryovials. These steps were completed within two hours of sample collection to maintain the integrity of the biological samples. No significant processing problems were experienced at any stage, thus blood and urine samples from all subjects were successfully collected and stored at −80 °C until further processing.

## Discussion

The ICONS Study was designed to elucidate potential immune-metabolic mechanisms underlying AIS. Here, we report on the establishment of the feasibility of clinical and laboratory procedures of the study.

This feasibility study has helped us revisit our project recruitment target of 80%. Detailed study procedure monitoring including consent procedures, instruments used for data collection, questionnaire completion rates, and biological sample collection and processing met or exceeded the pre-set targets.

### Study strengths, limitations, and adjustments to procedures based on initial data

This study has several strengths. The collaborations established with different clinical services to set up recruitment procedures were instrumental in assuring successful patient recruitment. The collection of muscle biopsies prior to cauterization and adrenaline injections preserved muscle macrophage phenotype and tissue integrity, thus allowing study questions to be addressed comprehensively, and established the feasibility of tissue sampling for the full study.

Study procedures and equipment used have proven to be highly reliable as predetermined feasibility criteria for participant consenting, sociodemographic data collection, resource utilization and sample acquisition and processing rates were all superseded. Furthermore, the validity of all measurement tools and questionnaires implemented have already been established [16, 17]. Finally, the study protocol places limited burden on the participants, as they are providing samples while undergoing surgery, so there are no added procedures to their routine care.

We achieved a lower than expected rate of participation due to scheduling challenges, but a significant number of those approached agreed to participate in the study.

Based on this data, we have designated one team member to be responsible for recruitment. This person is in charge of attending clinic visits to maximize the chances of a successful recruitment, and reduce the burden on the participants by doing all study recruitment and consenting procedures during the visit.

With these adjustments, the effect of further changes will take time to demonstrate effectiveness. We initially expected to approach around 40 patients per year based on estimated prior annual numbers of scoliosis surgeries at our center, but this was not the case during the feasibility study, with up to 30% of scoliosis cases requiring surgery being not AIS, in addition to the situations noted above [15].

Based on our estimate of approaching 40 patients in a year we predicted a 5-year recruitment period would suffice. This feasibility study is instrumental in highlighting the need for more generous timelines for recruitment. While there is merit to this approach, the delay in knowledge generated from this project can have a significant impact on its translation to interventions that benefit patients. In addition, the sustainability of the study funding is another potential caveat to this approach.

Alternatively, expanding the study to other pediatric orthopedic centers will help achieve our target sample size of 120 subjects. We have engaged in discussions with other centers to expand recruitment and address the sample size needed for the full study. One center is quite interested in joining the study, which will improve the recruitment timelines. There are planned discussions with other centers that are interested in getting more information about the study. The feasibility of this expansion needs further evaluation to ensure its feasibility.

One potential limitation of the ICONS study is its cross-sectional design, which makes investigating causal links of muscle-immune mechanisms to AIS development difficult to establish. However, there are no reliable experimental models beyond humans to study AIS [26, 27]. In addition, the potential molecules and pathways identified in this study can be validated further in cell culture and in vivo models as dictated by the results.

This study does not incorporate a control sample to compare muscle phenotype in AIS to non-AIS subjects. The inclusion of a control group is neither feasible nor ethically justified, as this would expose healthy children or those with less severe degrees of scoliosis to potential harms of invasive biopsy procedures. In addition, longitudinal study designs are also not appropriate, as we cannot implement multiple biopsies to measure muscle inflammation with curve progression over time, and repeated biopsies may have their own inflammatory response in muscle at sites of biopsy. Ultimately, our cross-sectional design is the most appropriate approach.

The study is susceptible to various sources of bias. Selection bias may occur if our sample was not representative of the pediatric population with AIS by including other types of scoliosis. This is likely to be limited, as our population of pediatric patients with AIS requiring surgery constitutes a representative group of patients with AIS.

Recall bias may also occur when participants are completing questionnaires that inquire about subjective and/or past lifestyle or family history-related factors. We attempted to minimize this bias by correlating self-reported data with clinically obtained data that was relevant to the immediate perioperative period.

Social desirability bias may also be a threat given the subjective nature of the lifestyle questionnaires. We utilized structured data collection tools that are previously reported to reflect the clinical phenotype of children accurately [16, 17, 23].

### Potential outcomes of our research

The novelty of the study offers significant implications to patients with AIS. Identifying potential mechanisms underlying AIS will create new knowledge that can be translated into interventions to prevent and treat AIS. This study may also guide further experimental work in cell-based and animal models to test specific pathways that mediate AIS. Collectively, this may allow the creation of interventions that can be tested to validate their efficacy and safety.

In addition, new insights to the complications of AIS may be gleaned from this experimental work, whereby new approaches to prevent these complications may emerge from understanding the pathogenesis of AIS, ensuring improved outcomes. Importantly, our results may develop novel insights into immune-metabolic crosstalk in AIS, which may be an important aspect of the etiopathogenesis of this condition.

Our work is also of importance in the field of translational research studies in pediatrics. AIS affect millions of children around the world, and causal factors are not fully understood. As paraspinal muscle provides spinal stability and directs spinal motion, it is critical that we understand the muscle- and immune-based mechanisms that may contribute to AIS. It is also important to inform the scoliosis research community about the potential for success of studies similar to ours, and to highlight the need for early consideration of sample size achievement. In addition, this paper will be of broader importance to colleagues performing translational research to describe approaches to study procedures and anticipated outcomes.

## Conclusion

We have determined that the protocols and procedures for the ICONS study are feasible. However, to overcome the lower than expected recruitment rates, we will consider the prolongation of the inclusion period and the expansion of the study to new centers. We will be moving with the implementation of these protocols taking these considerations into account.

## Abbreviations

AIS: Adolescent idiopathic scoliosis
BMI: Body mass index
ICONS: Immune-Metabolic CONnections to Scoliosis
mmHg: Millimeters of mercury
